# High-throughput metabolomics identifies new biomarkers for cervical cancer

**DOI:** 10.1007/s12672-024-00948-8

**Published:** 2024-03-29

**Authors:** Xue Li, Liyi Zhang, Xuan Huang, Qi Peng, Shoutao Zhang, Jiangming Tang, Jing Wang, Dingqing Gui, Fanxin Zeng

**Affiliations:** 1 Department of Clinical Research Center, Dazhou Central Hospital, Dazhou, 635000 Sichuan China; 2 Department of Gynaecology and Obstetrics, Dazhou Central Hospital, Dazhou, Sichuan China; 3 Department of Medical Research Center, Beijing Chaoyang Hospital, Capital Medical University, Beijing, China; 4 Department of Clinical Laboratory, Beijing Anding Hospital, Capital Medical University, Beijing, China

**Keywords:** Cervical cancer, Metabolites, Trimethylamine *N*-oxide, Prognosis

## Abstract

**Background:**

Cervical cancer (CC) is a danger to women’s health, especially in many developing countries. Metabolomics can make the connection between genotypes and phenotypes. It provides a wide spectrum profile of biological processes under pathological or physiological conditions.

**Method:**

In this study, we conducted plasma metabolomics of healthy volunteers and CC patients and integratively analyzed them with public CC tissue transcriptomics from Gene Expression Omnibus (GEO).

**Result:**

Here, we screened out a panel of 5 metabolites to precisely distinguish CC patients from healthy volunteers. Furthermore, we utilized multi-omics approaches to explore patients with stage I-IIA1 and IIA2-IV4 CC and comprehensively analyzed the dysregulation of genes and metabolites in CC progression. We identified that plasma levels of trimethylamine *N*-oxide (TMAO) were associated with tumor size and regarded as a risk factor for CC. Moreover, we demonstrated that TMAO could promote HeLa cell proliferation in vitro. In this study, we delineated metabolic profiling in healthy volunteers and CC patients and revealed that TMAO was a potential biomarker to discriminate between I-IIA1 and IIA2-IV patients to indicate CC deterioration.

**Conclusion:**

Our study identified a diagnostic model consisting of five metabolites in plasma that can effectively distinguish CC from healthy volunteers. Furthermore, we proposed that TMAO was associated with CC progression and might serve as a potential non-invasive biomarker to predict CC substage.

**Impact:**

These findings provided evidence of the important role of metabolic molecules in the progression of cervical cancer disease, as well as their ability as potential biomarkers.

**Supplementary Information:**

The online version contains supplementary material available at 10.1007/s12672-024-00948-8.

## Introduction

Cervical cancer (CC) is an important global public health issue, which ranks as one of the most common cancers and the top leading cause of cancer death in middle-aged women [1, 2]. CC has multiple risk factors including high-risk human papillomavirus (HPV) infection, unsanitary sexual behaviors, and long-term use of oral contraceptives [3]. Pap Smear and HPV tests are common screening procedures for cervical intraepithelial neoplasia (CIN) or CC [4, 5]. However, the gold standard for diagnosing and staging of CC depends on the tissue pathological characteristics of biopsy samples, which is obtained from an invasive pressure. According to the International Federation of Gynecology and Obstetrics (FIGO) guidelines, CC is divided into four stages, each of which contains, A and B sub-stages [6]. The precise and proper staging determination directly affects the treatment plans for CC patients [7].

Metabolomics can make the connection between genotypes and phenotypes [8]. It provides a wide spectrum profile of biological processes under pathological or physiological conditions. Small-molecule metabolites can not only act as biomarkers for disease conditions but also often participate in signal-transduction pathways to modulate phenotype [9]. The metabolomics-based screening is a robust and efficient way to discover candidate biomarkers for early cancer diagnosis, progression, and prognosis [10, 11]. Hasim et al. have performed plasma amino acid profiling in CC and CIN patients compared with healthy volunteers. It was found that CC and CIN patients had low levels of overall amino acid, and Lysine, tryptophan, cysteine and methionine metabolism pathways were also revealed differentially expressed in urine samples of CC patients in Liang et al.’s report [12, 13]. Yin et al. identified phosphatidylcholine and lysophosphatidylcholine in plasma acting as discriminate biomarkers to divide squamous cervical cancer (SCC) and uterine fibroid (UF) patients [14]. Yang et al. via integration of metabolomics and transcriptomics provided 5 candidate metabolites to diagnose CC [15]. Khan et al. reported that a panel of 7 metabolites can be used as early detection biomarkers to distinguish CIN and CC patients [16]. Moreover, stage 2 CC can be divided into two substages, 2A and 2B, according to the sites of metastasis. Especially, depending on the tumor size, stage 2A could be further divided into two subgroups, i.e. stage I-IIA1 and stage IIA2-IV. The preferred treatment for I-IIA1 patients is radical hysterectomy, while the radical hysterectomy plus chemoradiation is the preferred treatment for IIA2-IV patients. However, there is no non-invasive marker for distinguishing stages I-IIA1 and IIA2-IV.

Plasma concentration of trimethylamine *N*-oxide (TMAO) is associated with gut microbiota and diet [17]. After gut microbiota transforming l-carnitine and choline to trimethylamine (TMA), TMA is transported to the liver and further oxidized to TMAO primarily by flavin-containing monooxygenase 3 (FMO3) [18]. Meat and eggs are important sources of l-carnitine and choline, while fish contains plenty of TMAO. High levels of plasma TMAO are an important and independent risk factor for atherosclerosis and cardiovascular disease. TMAO induces vascular inflammation through activation of NF-kB signaling and NLRP3 inflammasome [19, 20]. High levels of TAMO are also correlated with the risk of colorectal cancer (CRC) and prostate cancer. However, the carcinogenic mechanism of TMAO remains to be discovered [21].

In the present study, we conducted plasma metabolomics of healthy volunteers and CC patients and integratively analyzed them with public CC tissue transcriptomics from Gene Expression Omnibus (GEO). We delineated the metabolic fingerprinting of CC patients and revealed 5 metabolites as biomarkers of CC through the least absolute shrinkage and selection operator (LASSO) model. We further divided CC patients into the I-IIA1 group and IIA2-IV group according to FIGO definitions and comprehensively analyzed the metabolic profiling with the help of The Cancer Genome Atlas (TCGA) transcriptome data. We noticed that one of the 5 metabolites, TMAO, could mark off two groups. As a result, we conducted targeted metabolomics to quantify plasma TMAO concentration in derivation and validation cohorts and verified the effectiveness of TMAO. Moreover, high TMAO levels in plasma indicated an increased risk of tumor growth, coincident with the experiment result that TMAO promoted HeLa cervical cancer cell proliferation in a dose-dependent manner.

## Methods

### Study participants and metabolites extraction

Plasma samples used in this study were collected from patients and healthy volunteers who visited Dazhou Central Hospital from September 2017 to October 2020. We recruited a total of 34 healthy volunteers and 93 CC patients. Patients were diagnosed as CC according to pathological diagnosis. Based FIGO cervical cancer treatment guidelines and Chinese cervical cancer guidelines 2022 edition, IIA1 is an important decision point in the clinical treatment of patients, we merged patients with I-IIA1 into I-IIA1 group, and merged patients with IIA2-IV into IIA2-IV group for further subgroup analysis.

Plasma samples from − 80 ℃ were slowly dissolved at 4 ℃. One hundred μL plasma samples were mixed with 400 μL pre-cooled methanol acetonitrile solution (1:1, v/v). The mixture was vortexed for 60 s, placed at − 20 ℃ for 1 h, and centrifuged at 4 ℃ for 20 min. The supernatant was freeze-dried and analyzed by liquid chromatography (Agilent 1290 Infinity LC System). The sample was separated using the Agilent 1290 Infinity LC Ultra High Performance Liquid Chromatography (UHPLC) HILIC column. Column temperature was 25 ℃, and the flow rate was 0.3 mL/min. The mobile phase consisted of A (water, 25 mM ammonium acetate and 25 mM ammonia) and B (acetonitrile). During the entire analysis process, the sample was placed in a 4 ℃ automatic sampler. The chromatographic column parameters are Waters ACQUITY UPLC BEH Amide 1.7 µm, 2.1 × 100 mm column and Waters ACQUITY UPLC HSS T3 1.8 µm. Quality control (QC) samples were obtained by randomly mixing the need-checking samples. After UHPLC separation, for targeted metabolomics, 5500 QTRAP (AB SCIEX) was used for mass spectrometry analysis in electrospray ionization (ESI) positive mode. As for untargeted metabolomics, Triple TOF 5600 (AB SCIEX) mass spectrometer was used for mass spectrometry analysis in ESI positive mode and negative mode.

### Metabolomics analysis

For targeted metabolomics, Multiquant was used to extract peak area and retention time. The concentration of compounds between the two groups was compared by unpaired Student’s t-test. For untargeted metabolomics, LC–MS (liquid chromatography-mass spectrometry) raw data were preprocessed using XCMS to align peak, correct retention time and extract peak area. Metabolite structure was identified according to mass matching (< 25 ppm) and secondary spectral matching based on a self-built database (apt biotech). Then annotated peaks were normalized via pareto scaling using SIMCA 14.1. MetaboAnalyst was performed on univariate statistical analysis (T-test), multivariate statistical analysis (Principal component analysis, PCA; Orthogonal partial least squares discriminant analysis, OPLS-DA), enrichment analysis, joint pathway analysis, and network explorer. The differentially expressed metabolites were selected with criteria of adjust p-value less than 0.05, variable importance for the projection (VIP) value above 1 and absolute foldchange more than 1. Spearman correlation analysis was calculated by Hmisc and corrplot in R 3.6.3.

### Transcriptomics analysis

Transcriptomics data of 24 normal people and 28 CC patients are from GEO (Gene Expression Omnibus) DataSets (Series GSE63514) and differential gene expression was analyzed using GEO2R. Differentially expressed genes (DEGs) were selected with criteria of p-value less than 0.05 and absolute foldchange larger than 1. Transcriptomics data of five I-IIA1 stage and eight IIA2-IV stage CC patients are from TCGA (The Cancer Genome Atlas) and differential gene expression was analyzed using DESeq2 in R 3.6.3. GSEA software was used to select significant gene sets and calculate normalized enrichment score (NES) and p-value.

### Regression analysis and AUC calculation

In this study, the “Caret” package of R 3.6.3 was used to randomly divide patients into training and validation sets in a 7:3 ratio. The random grouping method was used to ensure that each patient had the same chance of being divided into the training group or the test group. LASSO regression model with tenfold cross-validation was built to discriminate between healthy volunteers and CC patients. The optimal λ value was picked as 1 standard error (SE) of the minimum λ to prevent the model from overfitting. The optimal λ value corresponded to 5 metabolites. LASSO model using threefold cross-validation was built to discriminate I-IIA1 and IIA2-IV patients. The optimal λ value was also selected the minimum λ value. The optimal λ value was matched to one metabolite which was then fitted to General Linear Model (GLM). Regression models were analyzed by glmnet in R 3.6.3.

### Cutoff value and AUC calculation

The optimal cutoff value was calculated according to the ROC curve and Youden index analysis by SPSS. ROC curves were built to calculate AUC using pROC in R 3.6.3.

### Survival analysis

R package survival and survminer were used to compute the Kaplan–Meier survival estimate and to plot survival curves. A log-rank test was used to compare two groups and determine statistical significance.

### Cell culture

Hela cell line was from FuHeng BioLogy (FH0314). Cells were cultured in RPMI1640 (11875093, Gibco) supplemented with 10% FBS and penicillin–streptomycin (15140163, Gibco) at 37 ℃ under 5% CO_2_.

### Cell proliferation and cytotoxicity assay

The cell suspension was incubated in a 96-well for 48 h and serum-starved for 12 h. Subsequently, cells were exposed to 0, 50, 100, 200, 400 and 800 µM TMAO (317594, Sigma-Aldrich) for 48 h. CCK-8 assay (CK04, DOJINDO) and EdU assay (KeyGEN BioTECH, KGA311) were performed to measure cell proliferation. Ten microliters of CCK-8 solution were added and incubated for 2 h and then absorbance was measured at 450 nm. Cells were added 10 μM EdU, fixed with 4% paraformaldehyde for 15 min, and supplemented with 0.5% Triton X-100 for 20 min. After washing cells with 3% BSA solution, the Click-It reaction buffer was incubated for 30 min in the shade. Subsequently, Edu stained cells were re-stained Hoechst 33342 for 30 min. After that, cells were imaged by a florescence microscope (IX71, Olympus). Cytotoxicity LDH assay was performed according to the manufacturer’s instructions (CK12, DOJINDO). These experiments were repeated 3 times.

## Results

### Plasma metabolic profiling revealed distinct metabolic alterations in CC patients

**Fig. 1:**
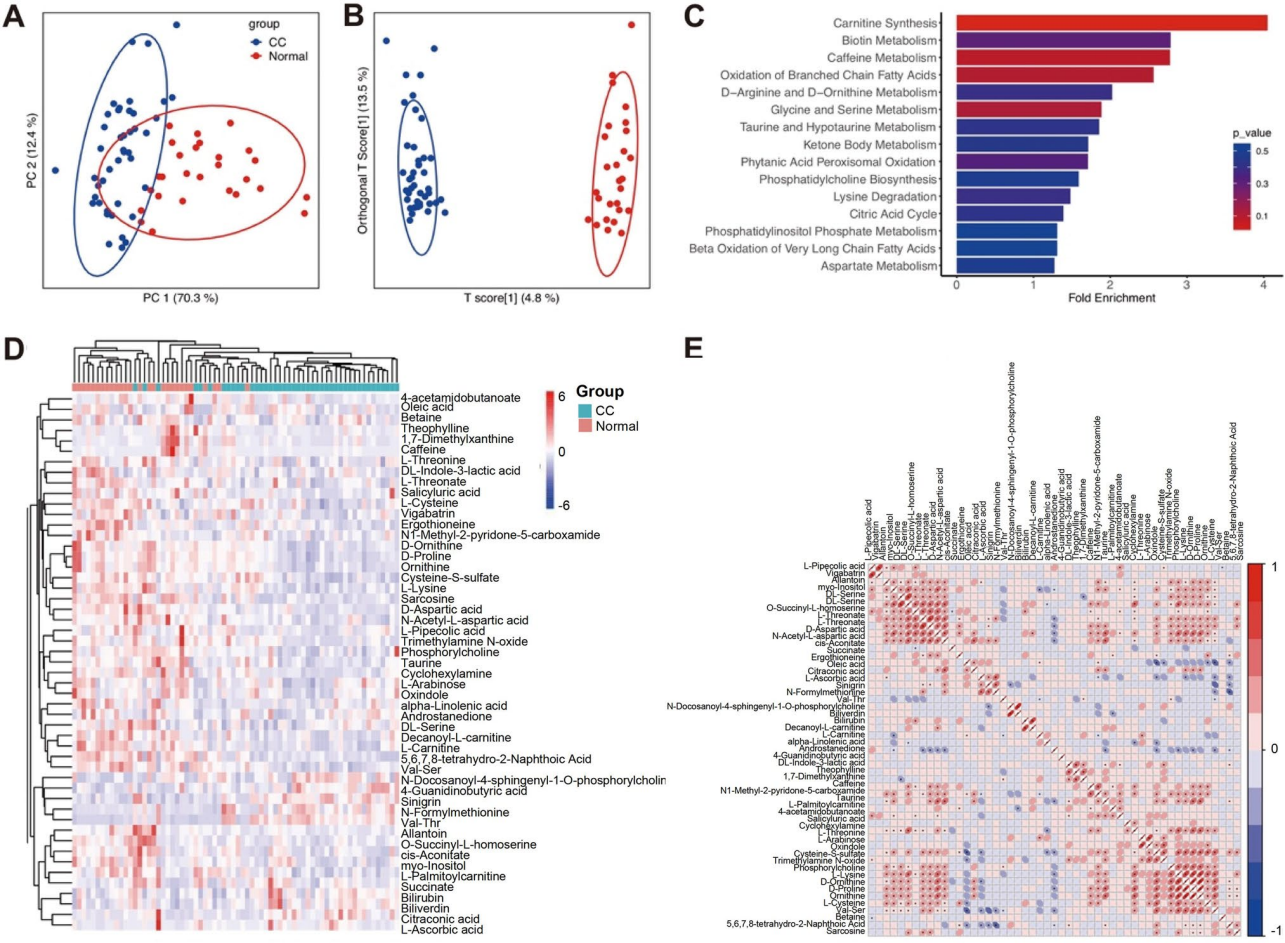
Metabolic molecular landscape in cervical cancer and control group. PCA (**A**) and OPLS-DA (**B**) score plot of metabolomics data. The ellipses display 95% confidence intervals. **C** Heatmap of differentially expressed metabolites using scaling peak intensity. **D** SMPDB (Small Molecule Pathway Database) enrichment analysis of altered metabolites. **E** Correlation plot of differentially expressed metabolites

We performed LC–MS-based untargeted metabolomics of plasma samples collected from 43 CC patients and 27 healthy volunteers, with the detailed information shown in Table S1. The flowchart of this study is shown in Figure S1. PCA was performed on metabolomics data from healthy volunteers and CC patients, with component 1 (PC1) for 70.3% and component 2 (PC2) for 12.4% of the variation (Fig. 1A). The orthogonal partial least-squares discriminant analysis (OPLS-DA) model was built by the aforementioned cohort showing a clear separation between the two groups. The OPLS-DA score plot in the predictive (x-axis) and orthogonal (y-axis) components were 4.8 and 13.5% respectively (Fig. 1B). Differential metabolomics analysis revealed 51 metabolites (Fig. 1C, Table. S2), and the differentially expressed metabolites were enriched in carnitine metabolism, lipid metabolism, and amino acid metabolism (Fig. 1D). Amino acid dysfunction has been reported in several studies. Multiple amino acids and their derivates appeared to coordinate with each other (Fig. 1E). In addition, we integrated transcriptomics from GEO DataSets including tissue specimens of 24 normal volunteers and 28 CC patients. The network of differential metabolites and genes indicated the source of the metabolites may result from abnormal tumor metabolism (Fig. S3).

### A panel of five metabolites serves as potential biomarkers for the diagnosis of CC

**Fig. 2:**
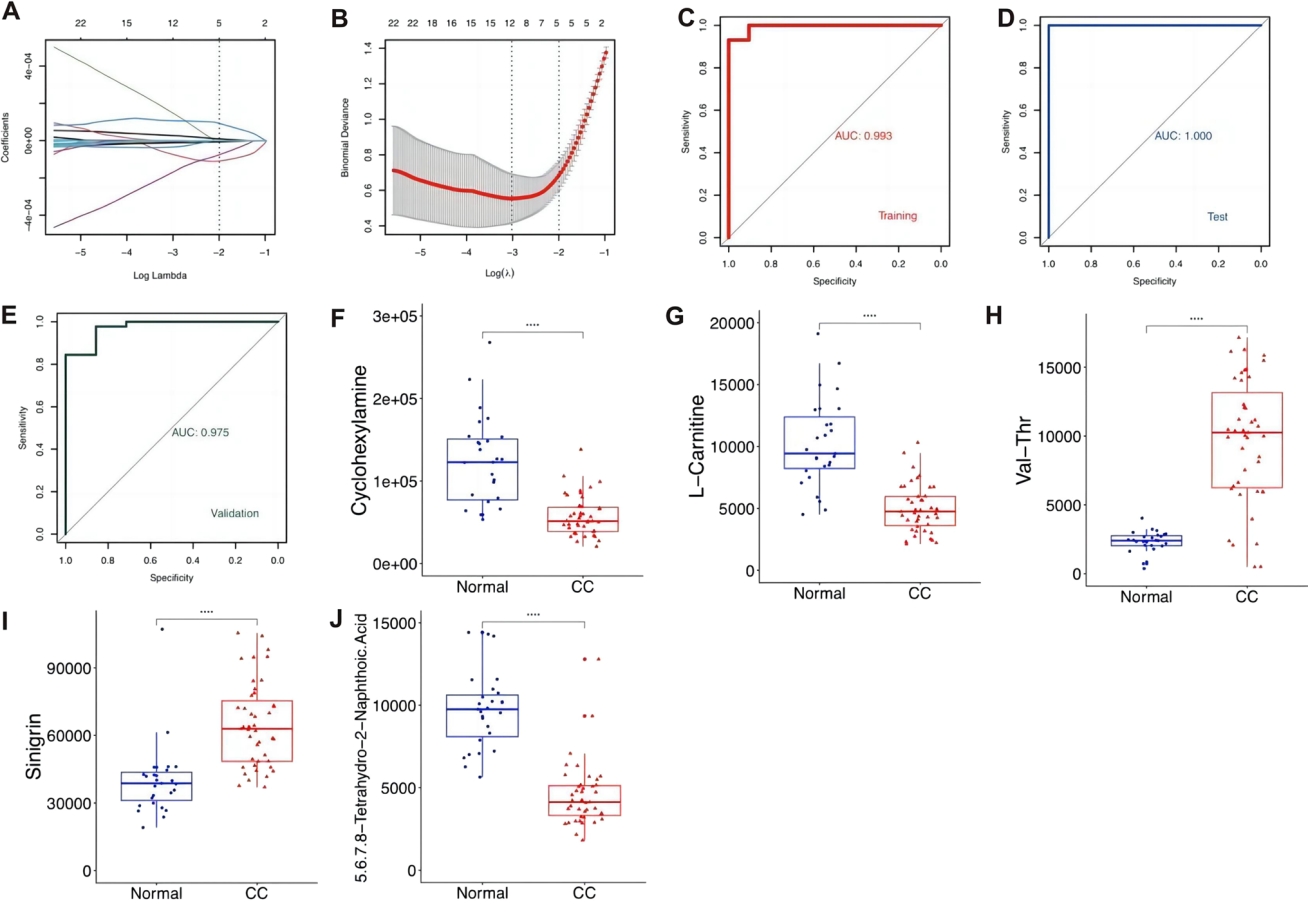
Identified metabolic biomarkers distinguishing cervical cancer from control. **A** Metabolites selection via LASSO regression analysis. ln(λ) is plotted on the x-axis while binomial deviance is plotted on the y-axis. **B** LASSO coefficient profiles of the 51 metabolites against ln(λ). **C** ROC curve for the training cohort. The AUC were 0.993 (95% CI 0.9808–1). **D** ROC curve for the test cohort. The AUC was 1 (95% CI 1–1). **E** ROC curve for the validation cohort. The AUC was 1 (95% CI 0.9279–1). **F**–**J** Boxplots of peak intensities of 5 potential biomarkers including cyclohexylamine, L-carnitine, Val-Thr, sinigrin, 5,6,7,8-Tetrahydro-2-Naphthoic acid with healthy volunteers and CC patients. The Student's *t-*test was used to evaluate the significance of the difference between the two groups

We performed tenfold cross-validation LASSO model with training cohort including 70% of the cohort to get the optimal λ value (right dotted line, lambda = 0.136) which was 1 standard error (SE) of the minimum λ (left dotted line, lambda = 0.049) (Fig. 2A). Five metabolites (Cyclohexylamine, l-Carnitine, Val-Thr, Sinigrin, 5,6,7,8-tetrahydro-2-Naphthoic acid) contributing to the model most were finally selected and combined to form a predictive model (Fig. 2B). Structures of the five metabolites were confirmed by standard compounds (Fig. S2). The 5-metabolite panel behaved equally well for the training cohort, test cohort, and another independent validation cohort including 45 CC patients and 7 normal people (Fig. 2C–E). The peak intensities of metabolites in the panel changed significantly between the two groups (Fig. 2F–J). A low concentration of l-Carnitine in plasma increases fatigue and exhaustion in cancer patients [22]. However, sinigrin, a natural product mainly from cruciferous vegetables which are reported to have anti-cancer therapeutic activity [23], unexpectedly rises in the plasma of CC patients. As a reason, we hypothesize that the dynamics of gut microbiota may influence the metabolism of sinigrin [24]. Further investigation into the association between three other metabolites and cancer is warranted.

### Multi-omics discrimination of I-IIA1 and IIA2-IV substages in CC

**Fig. 3:**
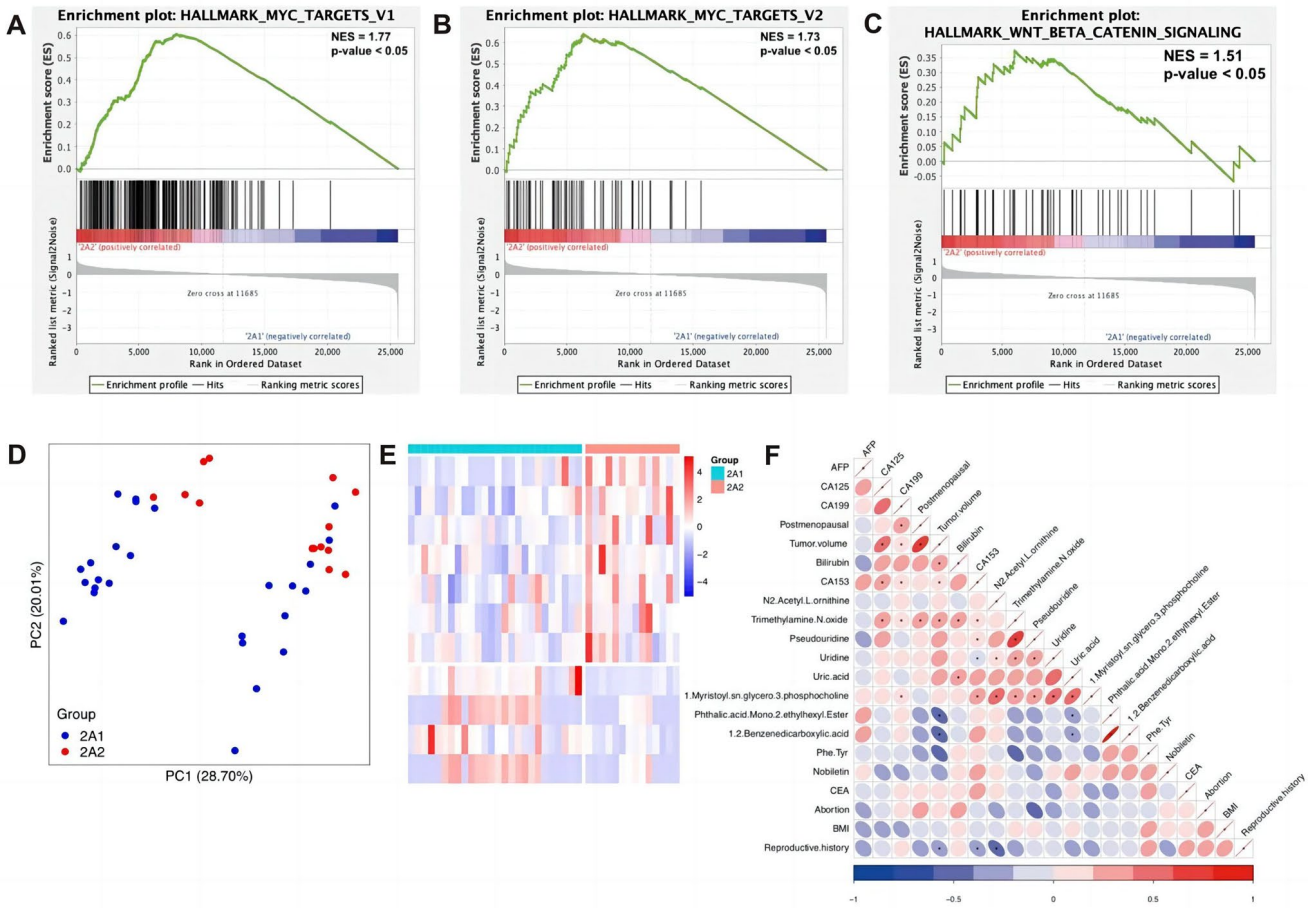
Differential metabolic landscape between I-IIA1 and IIA2-IV stages in cervical cancer. **A**–**C** GSEA analysis results of IIA2-IV compared to I-IIA1. **D**, **E** PCA score plot and heatmap of differentially expressed metabolites. **F** Correlation between differentially expressed metabolites and clinical indexes

According to FIGO staging criteria, I-IIA1 and IIA2-IV are divided based on the greatest dimension of tumor size. For the smaller tumor size, radical hysterectomy is more suitable for patients in stage I-IIA1 and prevents additional chemoradiotherapy. To distinguish the molecular profiles of patients both in I-IIA1 and IIA2-IV, we carried out homemade metabolomics and TCGA-derived transcriptomics analysis. Gene sets enrichment analysis (GSEA) showed terms related to tumor progression (Fig. 3A–C). *MYC* is an oncogene that promotes cell proliferation as a transcription factor and Wnt/β-catenin signaling is activated in cancers [25, 26]. Using 11 differentially expressed metabolites, PCA analysis was performed with PC1 and PC2 adding up to 48.71%. The heatmap illustrated different patterns of top 11 significantly changed metabolites between two groups. We performed correlation analysis of differentially expressed metabolites of clinical indexes and found that tumor volume was associated with several metabolites such as TMAO and cancer biomarkers CA125 (Fig. 3F).

### TMAO concentration reflects features of I-IIA1 and IIA2-IV patients as a biomarker

**Fig. 4:**
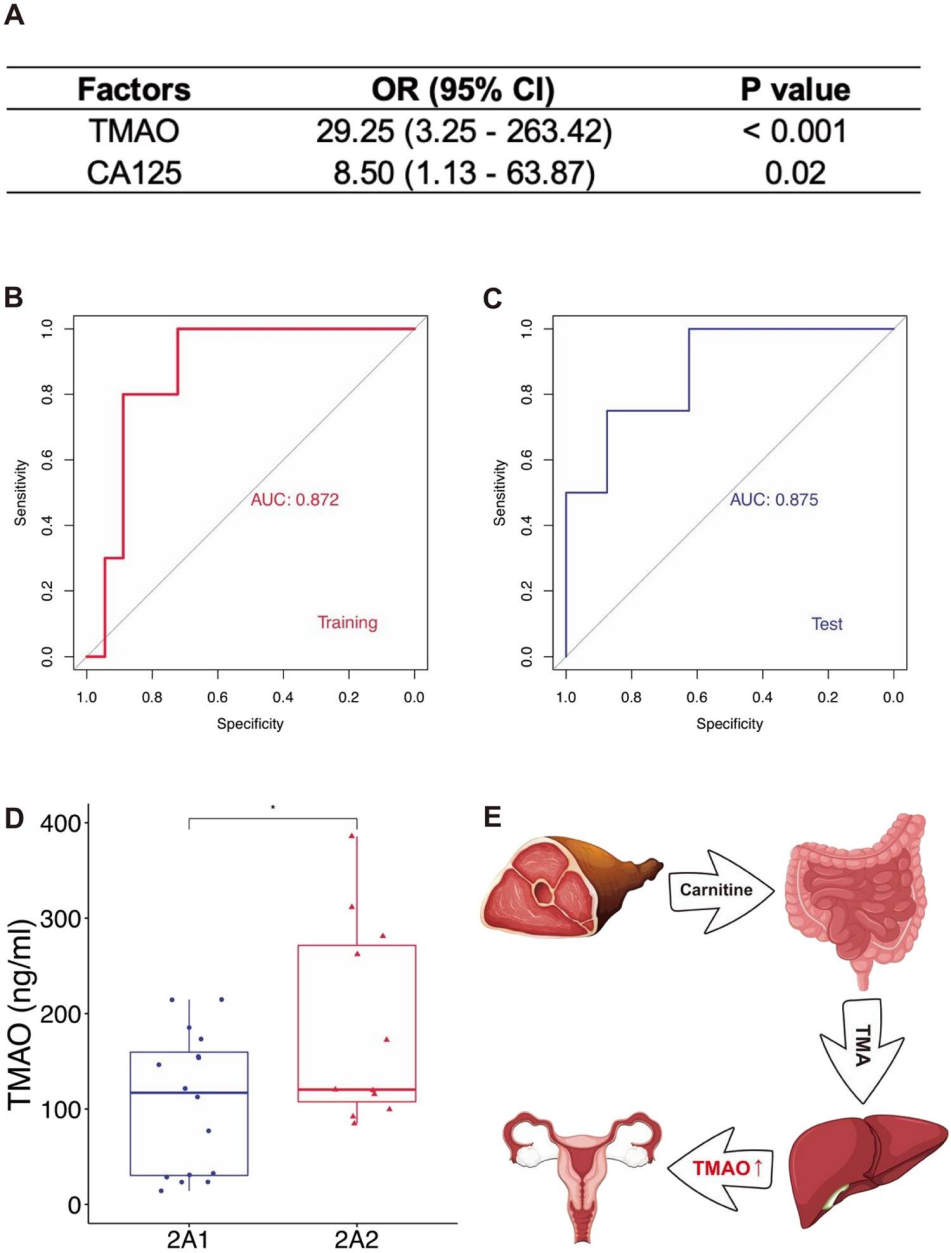
Identification of TMAO as a biomarker for different stages of cervical cancer patients. **A** Odds ratios with 95% CI and p-value, of TMAO and CA125 in predicting CC progression from I-IIA1 to IIA2-IV for all patients. **B** ROC curve of TMAO for the training cohort. The AUC was 0.872 (95% CI 0.7338–1). **C** ROC curve of TMAO for the test cohort. The AUC was 0.875 (95% CI 0.6578–1). **D** Boxplot of concentration of TMAO with I-IIA1 and IIA2-IV patients in derivation and validation cohorts. **E** Schematic diagram of sources of TMAO, showing increased TMAO and unchanged TMAO precursors in plasma of IIA2-IV patients compared to those of I-IIA1 patients

Concerning CC progression risk factors, we determined cutoff values based on the Youden index for TMAO and CA125 concentration as indicators. The cutoff value-based OR for classifying I-IIA1 and IIA2-IV indicated that TMAO was strongly correlated with CC progression while CA125 was weakly correlated (Fig. 4A). ROC curve analysis also served to confirm TMAO as a more accurate biomarker compared to CA125 on categorizing I-IIA1 and IIA2-IV patients (Fig. 4B, C and Fig. S4A, B). Survival analyses of I-IIA1 versus IIA2-IV show no significant differences in previous reports [27, 28]. However, in our cohort, stage IIA2-IV tended to represent a poor prognosis. Due to the small sample size, the statistical power may be not strong (Fig. S4C). We conducted targeted metabolomics on 27 plasma specimens. There was a significant increase in plasma concentration of TMAO in the IIA2-IV patients against the I-IIA1 patients (Fig. 4D, E and Fig. S5). Unexpectedly, the precursors of TMAO, carnitine, choline, and TMA, were not significantly changed between the two groups (Fig. S4D–F and Fig. 4E).

### TMAO enhanced cancer cell proliferation in vitro.

**Fig. 5:**
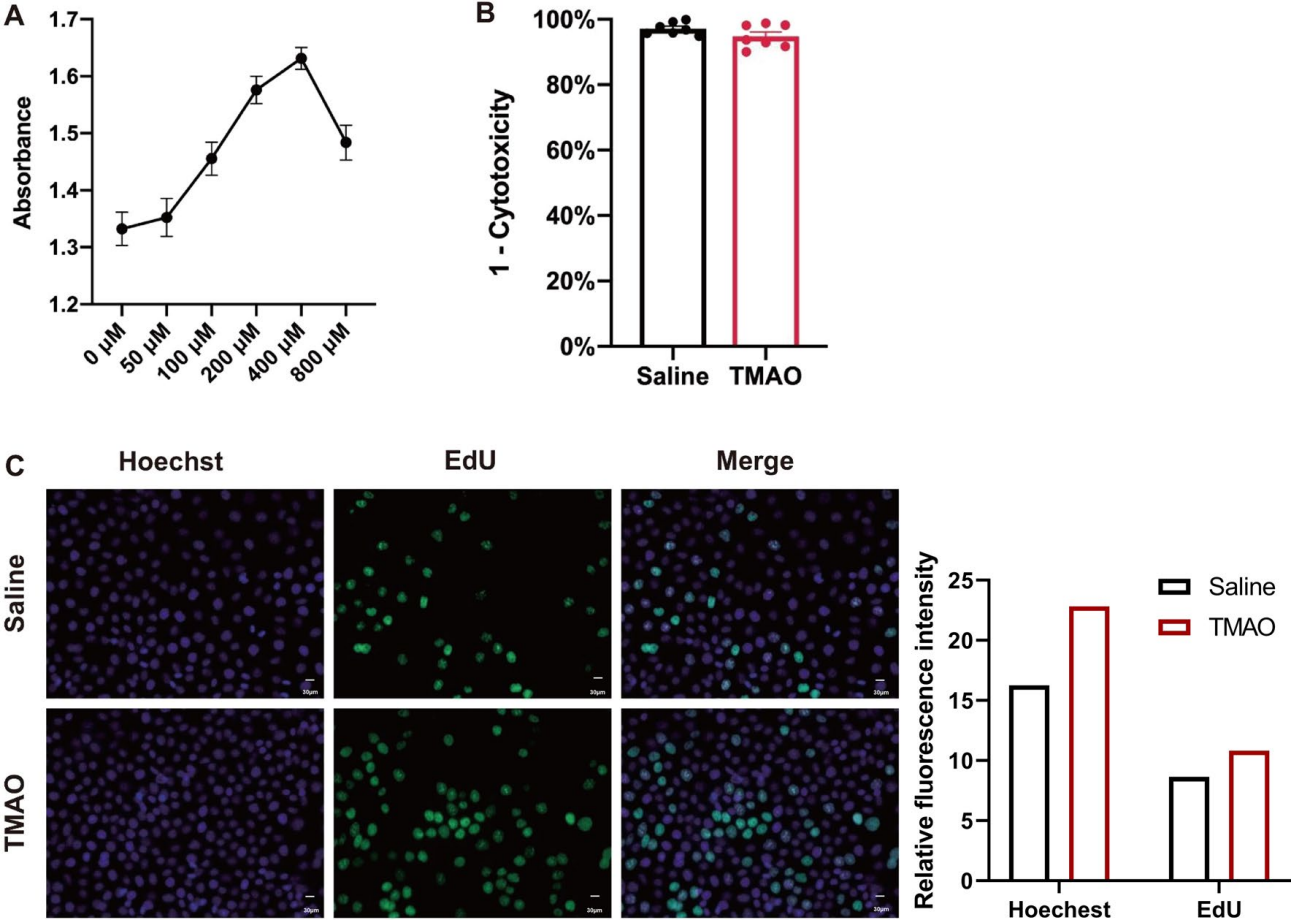
TMAO functional analysis in vitro. **A** TMAO promoted HeLa cell proliferation in a dose-dependent manner. **B** HeLa cells treated with 400 μM TMAO were tested for LDH assay to assess TMAO cytotoxicity. **C** HeLa cells treated with 400 μM TMAO were tested for EdU assay. Representative immunofluorescence images of Hoechst, EdU, and the merged

To further investigate the clinical significance of increased TMAO concentration in plasma, we conducted functional studies at the cellular level in vitro. TMAO does not affect promoting endothelial cell viability under a series of concentrations from 50 to 1000 μM at different time points [20]. Considering that cancer cells may respond to different growth-stimulating signals from normal cells [29], we performed a CCK8 assay to analyze the proliferation of HeLa cells following treatment with a gradient concentration of TMAO in the culture medium for 48 h. The result showed that TMAO could modulate proliferation in a dose-dependent manner with a peak capacity at 400 μM (Fig. 5A). Under 400 μM treatment for 48 h, HeLa cells high exhibited cell counts and EdU positive cells (Fig. 5C) without increasing damaged cells through measuring lactate dehydrogenase activity (Fig. 5B). These results together indicated that TMAO has proliferation stimulating activity in HeLa cells.

## Discussion

Sustaining proliferation and metabolic reprogramming are considered as hallmarks of cancer [30]. Metabolic disorders of tumor cells often induce abnormal metabolism of carbohydrates, lipids, amino acids, and other small molecules in cancer patients.

In this study, we mainly used metabolomics data to depict metabolic changes in onset and the progression of CC. We observed that the metabolism of a few core metabolites such as lipids and amino acids were altered in CC patients and the abnormal metabolic changes were accompanied by progression of CC. Due to its stable performance, LASSO is widely accepted and used to sort key variables and build clinical predictive models. Based on the differential metabolites we screened, we generated a diagnostic model consisting of five metabolites in plasma which was effective and non-invasive compared to the gold standard of histological diagnosis. The current study was limited to a single-center and has several other constraints. It should be acknowledged that the validation cohort exhibited an imbalance, which may not provide sufficient evidence for establishing a robust model.

As for the five metabolites, it remains unclear how these metabolites participate in the occurrence of CC and how metabolic changes in CC patients influence plasma metabolite abundance. However, there are some research reports on these 5 metabolic molecules in other diseases. Cyclohexylamine was identified as the serum metabolite biomarkers of the persons with type 2 diabetes with multiple complications [31]. l-carnitine was reported to decrease body weight and BMI through a variety of mechanisms, such as improving insulin resistance and may decrease appetite and food intake through a direct effect on hypothalamus [32–35]. Previous study showed that serum l-carnitine concentrations had a protective impact on overall, digestive system, and non-digestive system cancer risk [36]. Recent research supported decreases in Pro-CoA and its derivative propionyl-l-carnitine due to *ALDH6A1* downregulation were tightly associated with hepatocellular carcinoma [37]. The 5,6,7,8-tetrahydro-2-Naphthoic Acid has been proven to be a degradation product of Naphthalene [38] and there were no reports of its association with diseases. Palaniraja et al. found that Valine tRNA levels and availability regulate complex I assembly in leukemia [39], Sai et al. proposed that L-valine may be a potential marker for the diagnosis of lung cancer [40]. However, there is currently a lack of reports on Val-Thr in disease research. The sinigrin harnessed like a prodrug catalyzed by myrosinase to the production of AITC, which induced cell apoptosis and arrested the growth of lung cancer cells [41].

Based on FIGO guidelines, 2A patients were separated into two groups to make different plans of treatment. For I-IIA1 patients, we perform radical surgery without chemoradiotherapy is supposed. For IIA2-IV patients, we considered combining chemoradiotherapy and surgery to take control of patients’ condition. To our knowledge, we comprehensively compared the metabolic profiles of the two groups through metabolomics and transcriptomics, finding that TMAO is a valuable diagnostic biomarker for discriminating the two groups.

TMAO is a small molecular compound. After consuming food that is rich in carnitine and choline, in the presence of gut bacteria, these substances are converted to TMA and further oxidized to TMAO in the liver resulting in an increased plasma concentration of TMAO. A high concentration of TMAO is associated with an increased risk of cardiovascular diseases through promoting vascular inflammation. Seldin et al. and Chen et al. reported that TMAO induces vascular inflammation through different pathways, by activating NLRP3 inflammasome and NF-κB signaling respectively [19, 20]. In addition, some studies provided shreds of evidence that there is a link between TMAO and cancer, especially CRC [21]. In recent years, the role of TMAO in cancer has gradually been discovered. Recent researches identified the microbiome-derived metabolite TMAO drived immune activation and boosted responses to immune checkpoint blockade in pancreatic cancer [42], TMAO or its precursor choline, may represent a novel therapeutic strategy to promote the efficacy of immunotherapy in triple-negative breast cancer [43]. The Gut Microbial Metabolite TMAO has also been shown to promotes inflammatory hepatocellular carcinoma by upregulating POSTN [44].

Our study first proposed that TMAO is related to CC progression and may serve as a potential non-invasive biomarker to predict CC substages. However, we did not detect any changes in TMAO precursors in plasma. We speculate that it may be due to abnormal FMO3 activity, which converts TMA to TMAO in the liver or reduced TMAO excretion. Although the pathological mechanisms remain unclear, we had displayed that TMAO had a proliferation promotion effect on HeLa cells to unravel the puzzle.

### Supplementary Information

Supplementary file 1: Fig S1. Overview flowchart of the study. Fig S2. MS/MS spectra of cyclohexylamine (A), L-carnitine (B), Val-Thr (C), sinigrin (D), 5,6,7,8-tetrahydro-2-Naphthoic acid (E), (upper) matching to standard compounds (lower). Fig S3. Knitting network with both differentially expressed metabolites (blue nodes) and DEGs (red nodes). Fig S4. The performance of TMAO is to distinguish between I-IIA1 and IIA2-IV stages. (A) ROC curve of CA125 for the training cohort. The AUC was 0.821 (95% CI: 0.6231-1). (B) ROC curve of CA125 for the test cohort. The AUC was 0.750 (95% CI: 0.2132-1). (C) Survival plot and risk table of I-IIA1 (blue) and IIA2-IV (red) group. TMAO levels were determined by cutoff value. P value of the log-rank test was 0.015. (D - F) Boxplots of concentration of carnitine, choline, and TMA with I-IIA1 and IIA2-IV patients in derivation and validation cohorts. Fig S5. The expression level of TMAO in different pathological stages.

Supplementary file 2: Table S1 Characteristics of cervical cancer patients. Table S2 The 51 Differential metabolites between cervical cancer and control group. Table S3 Performance of two model in training, test and validation sets.

## Data Availability

The data that support the findings of this study are available from the corresponding author upon request.
