# Supplementary material for: High-throughput metabolomics identifies new biomarkers for cervical cancer
Source: Discov Oncol. 2024 Mar 29;15:90. doi: 10.1007/s12672-024-00948-8 (PMC10980666; doi:10.1007/s12672-024-00948-8)
Supplement: Supplementary file 1 — Fig S1. Overview flowchart of the study. Fig S2. MS/MS spectra of cyclohexylamine (A), L-carnitine (B), Val-Thr (C), sinigrin (D), 5,6,7,8-tetrahydro-2-Naphthoic acid (E), (upper) matching to standard compounds (lower). Fig S3. Knitting network with both differentially expressed metabolites (blue nodes) and DEGs (red nodes). Fig S4. The performance of TMAO is to distinguish between I-IIA1 and IIA2-IV stages. (A) ROC curve of CA125 for the training cohort. The AUC was 0.821 (95% CI: 0.6231-1). (B) ROC curve of CA125 for the test cohort. The AUC was 0.750 (95% CI: 0.2132-1). (C) Survival plot and risk table of I-IIA1 (blue) and IIA2-IV (red) group. TMAO levels were determined by cutoff value. P value of the log-rank test was 0.015. (D - F) Boxplots of concentration of carnitine, choline, and TMA with I-IIA1 and IIA2-IV patients in derivation and validation cohorts. Fig S5. The expression level of TMAO in different pathological stages. [file 12672_2024_948_MOESM1_ESM.pdf]

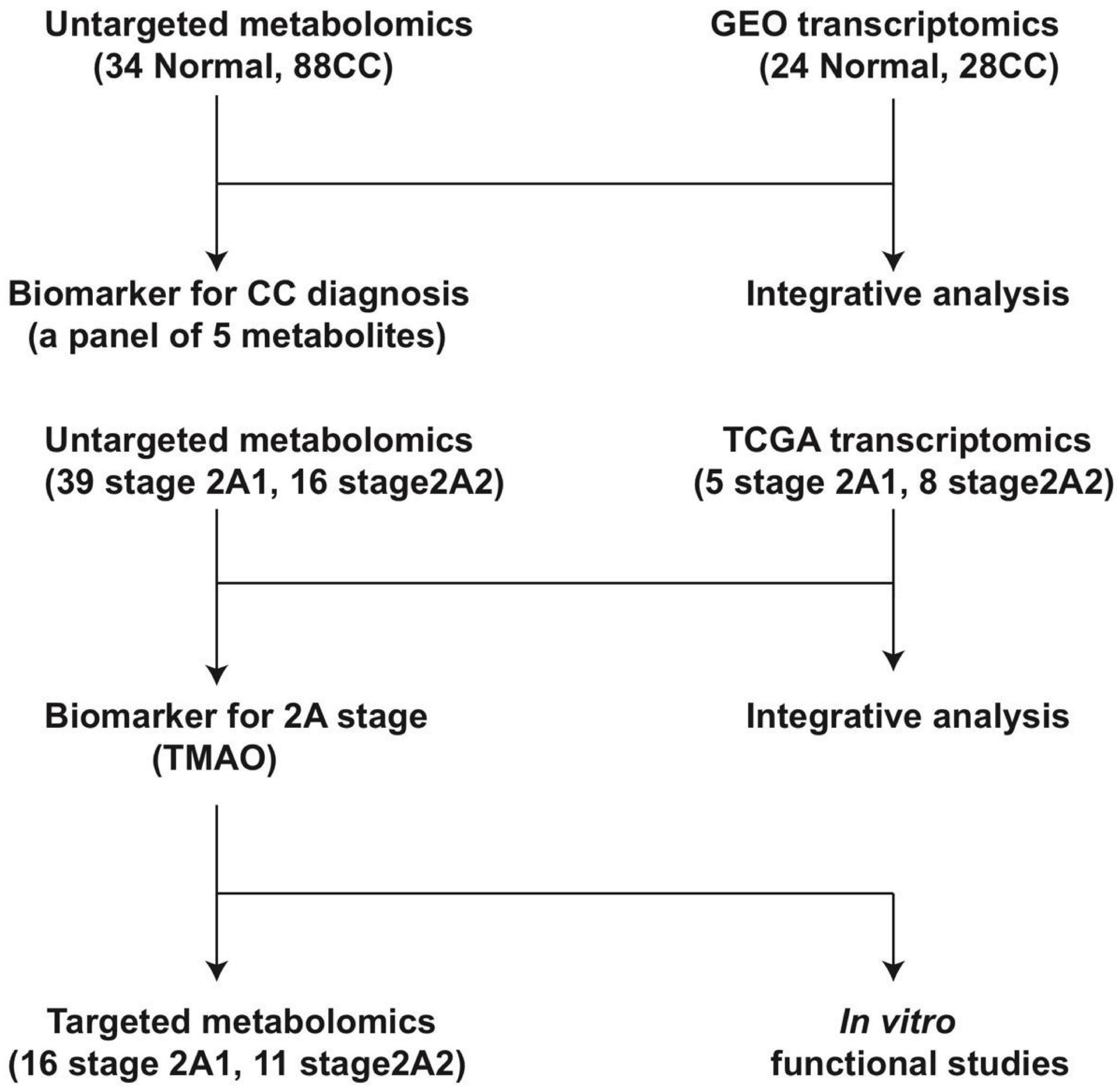

Fig S1. Overview flowchart of the study.

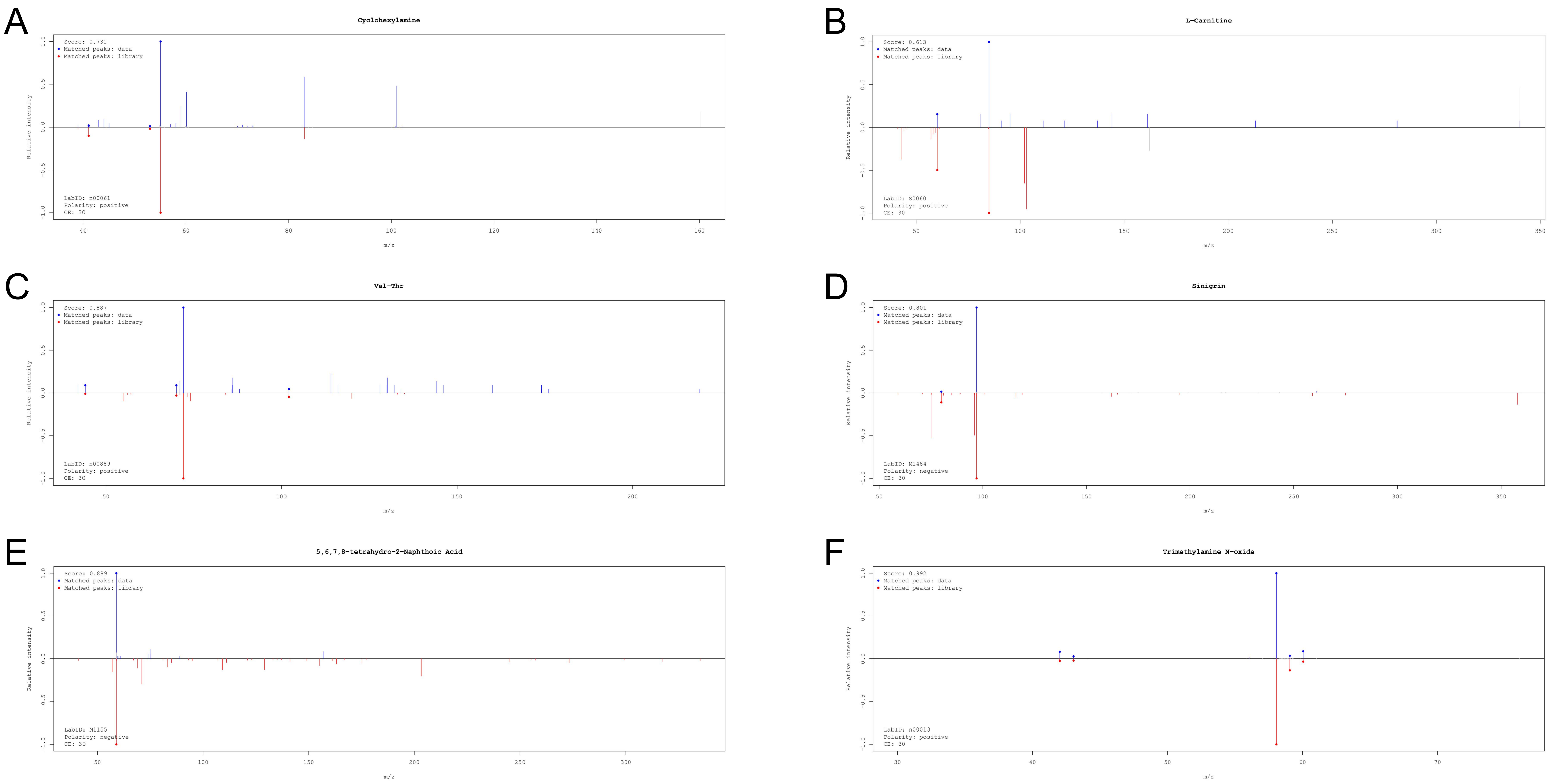

Fig S2. MS/MS spectra of cyclohexylamine (A), L-carnitine (B), Val-Thr (C), sinigrin (D), 5,6,7,8-tetrahydro-2-Naphthoic acid (E), (upper) matching to standard compounds (lower).

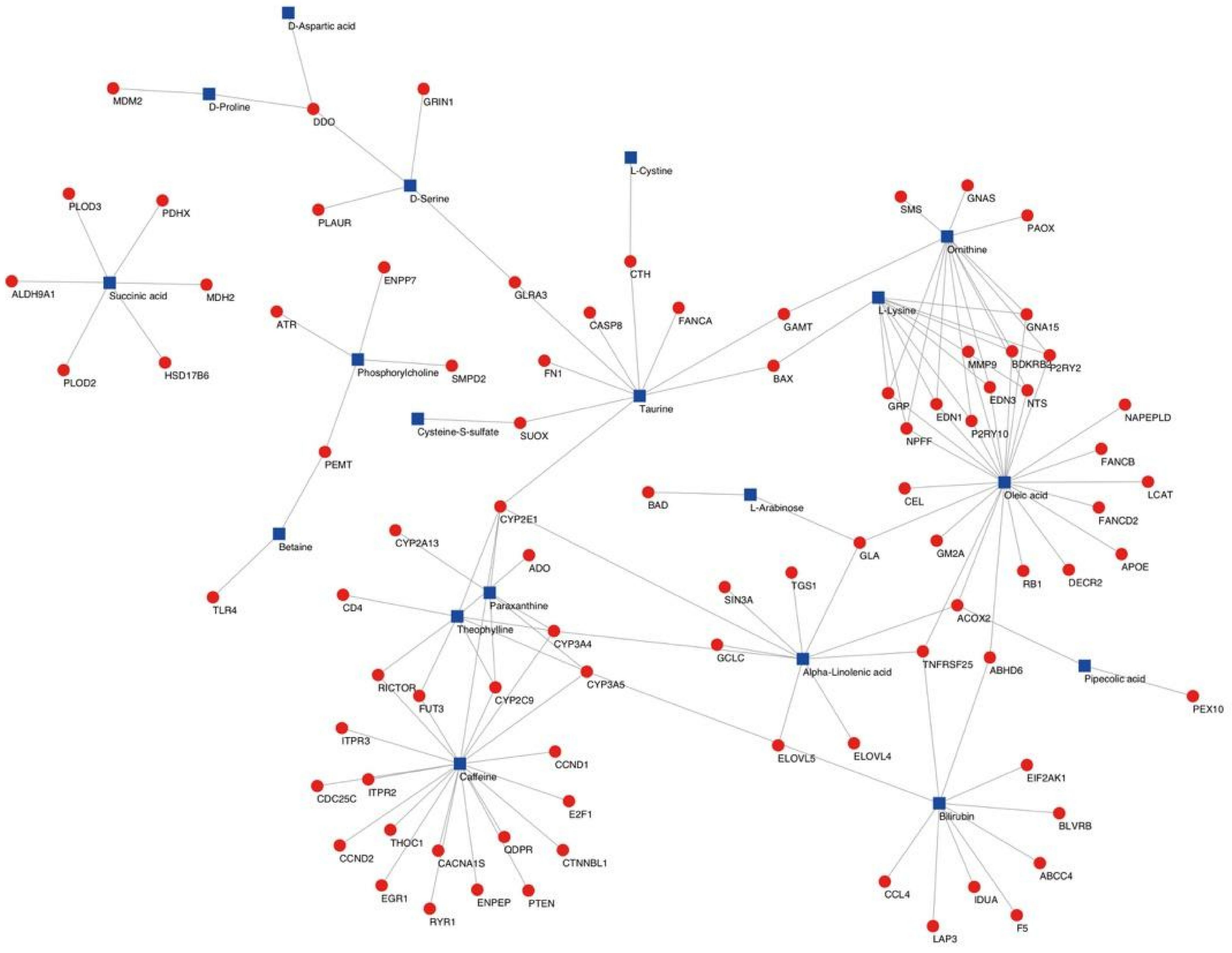

Fig S3. Knitting network with both differentially expressed metabolites (blue nodes) and DEGs (red nodes).

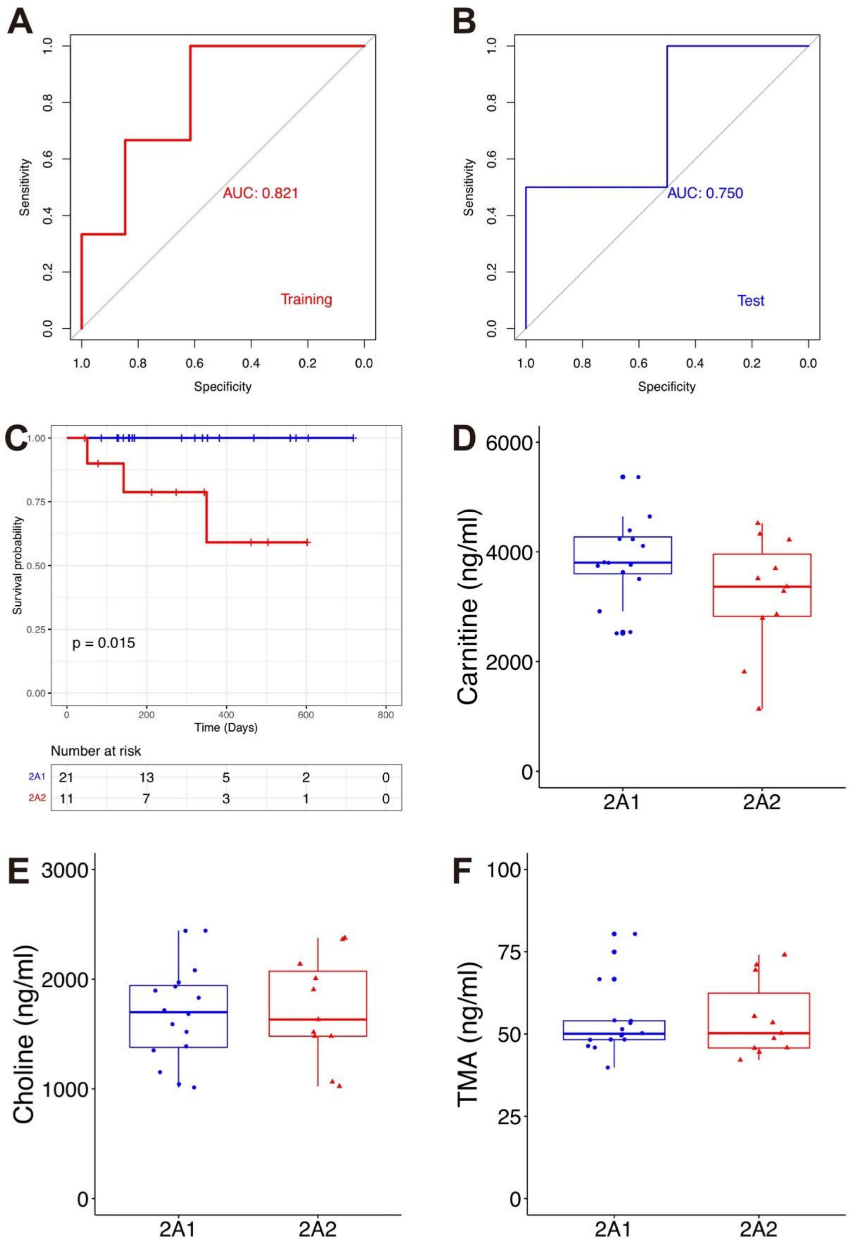

Fig S4.The performance of TMAO is to distinguish between 2A1 and 2A2 stages.

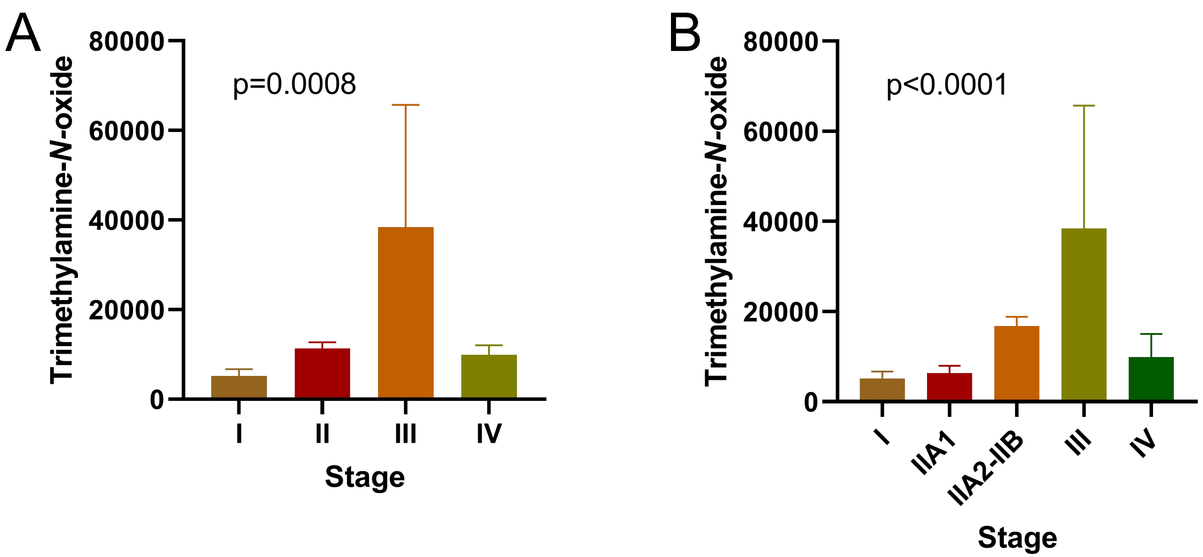

Fig S5.The expression level of TMAO in different pathological stages.
