# Supplementary material for: High-throughput metabolomics identifies new biomarkers for cervical cancer
Source: Discov Oncol. 2024 Mar 29;15:90. doi: 10.1007/s12672-024-00948-8 (PMC10980666; doi:10.1007/s12672-024-00948-8)
Supplement: Supplementary file 2 — Table S1 Characteristics of cervical cancer patients. Table S2 The 51 Differential metabolites between cervical cancer and control group. Table S3 Performance of two model in training, test and validation sets. [file 12672_2024_948_MOESM2_ESM.docx]

**Supplemental Information**

**Table S1 Characteristics of cervical cancer patients.**

|  | **Level** | **CC** | **Health** |
| --- | --- | --- | --- |
| N |  | 93 | 34 |
| Age (mean (SD)) |  | 53.62(10.19) | 52.21(9.84) |
| Education (%) | No information | 8(8.6) | 0(0.0) |
|  | ≤6y | 53(57.0) |  |
|  | 6-12y | 31(33.3) |  |
|  | ≥12y | 1(1.1) |  |
| Living condition (%) | No information |  |  |
|  | Rural area | 61(65.6) | 4(118) |
|  | Urban area | 30(32.3) | 30(88.2) |
| BMI (mean (SD)) |  | 24.03(5.21) | 23.55(2.65) |
| Reproductive. History (%) | No information | 1(1.1) | 0(0.0) |
|  | 0 | 2(2.2) | 0(0.0) |
|  | 1 | 19(20.4) | 25(73.5) |
|  | 2 | 38(40.9) | 7(20.6) |
|  | 3 | 21(22.6) | 1(2.9) |
|  | ≥4 | 12(12.9) | 1(2.9) |
| Abortion (%) | No information | 1(1.1) | 0(0.0) |
|  | 0 | 32(34.4) | 17(50.0) |
|  | 1 | 13(14.0) | 6(17.6) |
|  | 2 | 23(24.7) | 8(23.5) |
|  | 3 | 9(9.7) | 1(2.9) |
|  | ≥4 | 15(16.1) | 2(5.9) |
| Postmenopausal (%) | No information | 2(2.2) | 0(0.0) |
|  | No | 30(32.3) | 26(76.5) |
|  | Yes | 61(65.6) | 8(23.5) |
| Smoking status (%) | No information | 1(1.1) | 0(0.0) |
|  | No | 92(98.9) | 34(100.0) |
| SP (mean (SD)) |  | 126.07(19.42) |  |
| DP (mean (SD)) |  | 76.59(11.87) |  |
| Staging (%) | CIN3 | 2(2.2) |  |
|  | IA | 2(2.2) |  |
|  | IB | 3(3.2) |  |
|  | IB1 | 10(10.8) |  |
|  | IB2 | 1(1.1) |  |
|  | IIA | 5(5.4) |  |
|  | IIA1 | 33(35.5) |  |
|  | IIA2 | 14(15.1) |  |
|  | IIB | 14(15.1) |  |
|  | III | 1(1.1) |  |
|  | IIIB | 2(2.2) |  |
|  | IV | 3(3.2) |  |
|  | Terminal | 3(3.2) |  |
| Tumor volume (mean (SD)) |  | 36.17(33.90) |  |
| Tumor types (%) |  | 22(23.7) |  |
|  | Adenocarcinoa | 5(5.4) |  |
|  | Adenosquamos carcinoma | 2(22) |  |
|  | Squamous cell carcinomas | 64(68.8) |  |
| CEA (mean (SD)) |  | 3.31(3.65) |  |
| AFP (mean (SD)) |  | 3.21(2.39) |  |
| CA125(mean (SD)) |  | 25.37(28.64) |  |
| CA153(mean (SD)) |  | 14.39(11.10) |  |
| CA199(mean (SD)) |  | 26.02(83.21) |  |
| HPV (%) |  | 33(35.5) | 0(0.0) |
|  | Negative | 28(30.1) | 34(100.0) |
|  | Positive | 32(34.4) | 0(0.0) |

**Table S2 The 51 Differential metabolites between cervical cancer and control group.**

| Compound | Fold change | Adjust Pvalue | *m/z* | vip |
| --- | --- | --- | --- | --- |
| 1,7-Dimethyxanthine | 2.05 | 0.020 | 181.07 | 4.07 |
| 4-acetamidobutanoate | 2.06 | 0.005 | 146.08 | 1.14 |
| 4-Guanidinobutyric acid | 0.74 | 0.00063 | 146.09 | 1.02 |
| 5,6,7,8-tetrahydro-2-Naphthoic Acid | 2.15 | <0.00001 | 157.06 | 1.37 |
| Allantoin | 1.72 | 0.027 | 157.04 | 1.74 |
| Alpha-Linolenic acid | 1.46 | 0.00141 | 279.23 | 4.64 |
| Androstanedione | 1.36 | 0.032 | 271.21 | 1.07 |
| Betaine | 1.37 | 0.018 | 118.09 | 2.25 |
| Bilirubin | 1.72 | 0.015 | 585.27 | 2.09 |
| Caffeine | 3.82 | 0.015 | 195.09 | 2.11 |
| Cis-Aconitate | 1.28 | 0.031 | 173.01 | 1.29 |
| Citramalic acid | 1.51 | 0.00366 | 207.05 | 2.12 |
| Cyclohexylamine | 2.14 | <0.00001 | 160.13 | 4.07 |
| Cysteine-S-sulfate | 1.62 | <0.00001 | 201.98 | 1.75 |
| D-Aspartic acid | 1.80 | <0.00001 | 132.03 | 1.62 |
| Decanoyl-L-carnitine | 2.06 | <0.00001 | 316.25 | 3.40 |
| DL-Indole-3-lactic acid | 1.27 | 0.019 | 188.07 | 2.13 |
| DL-Serine | 1.31 | 0.00214 | 104.04 | 1.05 |
| D-Ornithine | 1.64 | <0.00001 | 115.09 | 1.14 |
| D-Proline | 1.61 | <0.00001 | 116.07 | 1.80 |
| Ergothioneine | 1.91 | <0.00001 | 230.10 | 1.33 |
| L-Arabinose | 1.5 | 0.00044 | 133.05 | 1.11 |
| L-Ascorbic acid | 1.45 | 0.021 | 175.02 | 1.17 |
| L-Carnitine | 2.06 | <0.00001 | 340.25 | 1.57 |
| L-Cystine | 1.33 | 0.00410 | 239.02 | 1.08 |
| L-Lysine | 1.27 | 0.00083 | 147.11 | 1.54 |
| L-Palmitoylcarnitine | 1.24 | 0.028 | 422.33 | 1.28 |
| L-Pipecolic acid | 1.41 | 0.015 | 130.09 | 1.69 |
| L-Threonate | 1.42 | <0.00001 | 135.03 | 1.07 |
| L-Threonine | 1.27 | 0.033 | 118.05 | 1.08 |
| Myo-inositol | 1.27 | 0.00675 | 179.05 | 1.12 |
| N1-Methyl-2-pyridone-5-carboxamide | 1.32 | 0.028 | 153.06 | 1.05 |
| 1. Acetyhl-aspartic acid | 1.49 | 0.00108 | 174.04 | 1.10 |
| N-Docosanoyl-4-sphingenyl-1-0-phosphorylcholine | 1.68 | 0.034 | 850.67 | 1.04 |
| N-Formylmethionine | 1.38 | <0.00001 | 219.08 | 1.25 |
| Oleic acid | 1.60 | 0.031 | 281.25 | 1.03 |
| D-Ornithine | 1.69 | <0.00001 | 115.09 | 1.15 |
| O-Succinyl-L-homoserine | 1.38 | 0.013 | 200.05 | 1.4 |
| Oxindole | 1.62 | 0.0026 | 134.06 | 1.29 |
| Phosphorylcholine | 1.32 | 0.0053 | 184.07 | 1.04 |
| Salicyluric acid | 1.83 | 0.0108 | 194.05 | 1.49 |
| Sarcosine | 1.77 | <0.00001 | 131.08 | 1.22 |
| Sinigrin | 1.62 | <0.00001 | 359.04 | 3.48 |
| Succinate | 1.80 | 0.0202 | 117.02 | 1.28 |
| Taurine | 1.25 | 0.0019 | 126.02 | 1.03 |
| Theophylline | 1.94 | 0.0415 | 179.05 | 1.16 |
| Trimethylamine N-oxide | 1.88 | 0.0101 | 76.08 | 1.79 |
| Val-Ser | 1.68 | <0.00001 | 205.12 | 1.88 |
| Val-Thr | 1.23 | <0.00001 | 219.13 | 1.50 |
| Vigabatrin | 1.47 | 0.0022 | 130.09 | 1.58 |

Note: VIP, variable importance for the projection.

**Table S3 Performance of Two model in Training, Test and Validation sets.**

|  | Training set | | Test set | | Validation set |
| --- | --- | --- | --- | --- | --- |
|  | Five makers | TMAO | Five makers | TMAO | Five makers |
| AUC | 0.993 | 0.872 | 1 | 0.875 | 0.975 |
| Specificity | 1 | 0.821 | 1 | 0.804 | 0.961 |
| Sensitivity | 1 | 0.928 | 1 | 0.913 | 1 |
| Accuracy | 0.988 | 0.856 | 1 | 0.812 | 0.944 |
| Balanced Accuracy | 0.983 | 0.812 | 1 | 0.806 | 0.985 |
